# Evaluation of the Efficacy of Metformin in the Treatment of Acne Vulgaris and Its Effects on Serum Lipid Metabolism

**DOI:** 10.1111/jocd.70497

**Published:** 2025-10-15

**Authors:** Jie Hu, Yuxin He, Peiyang Du, Yongqiong Deng, Xia Xiong, Yaxin Huang

**Affiliations:** ^1^ Department of Dermatology The Affliated Hospital of Southwest Medical University Luzhou Sichuan China; ^2^ Department of Dermatology Chengdu Integrated TCM & Western Medicine Hospital Chengdu Sichuan China

**Keywords:** acne vulgaris, insulin resistance, LC–MS analysis, lipid metabolomics, metformin

## Abstract

**Background:**

Acne vulgaris (AV) is a common inflammatory skin disease during adolescence. Metformin (MET) has recently been found to have the effects of regulating lipid disorders, suggesting its potential benefits in the treatment of AV patients.

**Method:**

Recruited 18 patients with moderate to severe AV, and then received MET treatment (AVM group) for a continuous period of 12 weeks, while 20 healthy controls (HC group) served as the control group. The Global Acne Grading System (GAGS) score and VISIA‐CRTM imaging system were used to evaluate the severity of AV patients before and after treatment, and the serum lipid metabolomics differences were detected by liquid chromatograph mass spectrometer (LC–MS) before and after treatment. Multivariate statistical analysis of differentially expressed lipid metabolites was performed using partial least squares discriminant analysis (PLS‐DA) and orthogonal partial least squares discriminant analysis (OPLS‐DA). The Mann–Whitney *U* test was used to analyze the differences in lipid metabolites between groups. Spearman correlation analysis was conducted to examine the correlation between differentially expressed serum lipid metabolites and the acne severity index. The Kyoto Encyclopedia of Genes and Genomes (KEGG) database was used to predict the metabolic pathways involved in the differentially expressed lipid metabolites in the AVM group.

**Results:**

Compared to before treatment, the GAGS score (*p* < 0.001), red zone (*p* < 0.001) and Porphyrin (*p* < 0.01) indices of AV patients significantly improved after oral administration of MET. The results of PLS‐DA and OPLS‐DA indicated a clear separation in the composition of lipid metabolites between AV patients and the HC group; however, after MET treatment, the composition of lipid metabolites in AV patients showed a trend towards resembling that of the HC group. The 25 lipid metabolites with the most significant differences between AV patients and the HC group were all restored to the levels of the HC group after MET treatment. The Spearman correlation results showed that the serum PC (16:1/22:6) concentration in AV patients before treatment was positively correlated with the porphyrin area index (*r* = 0.47, *p* = 0.049). The KEGG analysis revealed 6 metabolic pathways that showed significant downregulation after treatment with MET.

**Conclusion:**

The therapeutic effect of MET on patients with moderate to severe AV may be achieved through the positive regulation of lipid metabolism. Its molecular mechanism may be related to the downregulation of inflammatory mediators associated with choline metabolism and arachidonic acid metabolism.

## 1 Introduction

Acne vulgaris (AV) is an inflammatory skin condition that affects the follicular sebaceous gland unit. Although its pathogenesis is not yet fully understood, it is traditionally believed that the onset of AV is primarily due to a combination of various factors, including the colonization of *Cutibacterium acne*, excessive sebum secretion, the release of inflammatory mediators, and hormonal imbalances [1]. In addition, it has also been reported that elevated levels of serum total cholesterol (TC), triacylglycerol (TG), low‐density lipoprotein (LDL), and other lipid metabolism‐related molecules in patients with AV. The disruption of serum lipid metabolism is also considered to be one of the important reasons for the occurrence and development of AV [2, 3]. As AV often results in a detrimental appearance [4], patients are increasingly concerned about the effectiveness of anti‐acne medications. However, the first‐line treatment drug, isotretinoin, carries potential side effects and adverse drug reactions, necessitating a careful evaluation of its efficacy versus safety in clinical treatment decisions. Therefore, it is of significant clinical importance to explore safer treatment methods or adjunctive medications. Metformin (MET), a first‐line medication for the treatment of type 2 diabetes, has recently been found to positively impact insulin resistance [5] and regulate lipid disorders [6, 7]. In addition, there is evidence that MET can reduce androgen production, making it an alternative treatment for androgen secretion component [8, 9]. These pharmacological mechanisms of MET have shown that it may be beneficial for the treatment of AV. It is worth noticing that the efficacy of MET in AV patients without insulin resistance has also been confirmed by relevant clinical studies; however, its underlying mechanism has not yet been fully elucidated [10, 11, 12]. Consequently, this study aims to utilize liquid chromatography–tandem mass spectrometry (LC–MS) to conduct serum lipidomics analysis on a healthy controls (HC group) population and AV patients before and after metformin treatment, in order to investigate whether MET can provide an auxiliary therapeutic effect on AV patients by regulating lipid metabolism.

## 2 Method

### 2.1 Objective

From June 2019 to April 2020, 18 patients with moderate to severe AV who met the following criteria were recruited from the outpatient department of the Affiliated Hospital of Southwest Medical University. Inclusion criteria: patients diagnosed with moderate to severe AV; ages between 18 and 30 years; body mass index (BMI) between 18 and 25 kg/m^2^; no history of using isotretinoin or metformin in the past 6 months; no systemic use of any medication in the past 6 months. Exclusion criteria: other skin diseases, such as atopic dermatitis, eczema, psoriasis, etc.; systemic diseases, such as severe heart, liver, or kidney conditions; diabetes mellitus and malignant tumors; pregnant or breastfeeding women; individuals with smoking or alcohol addiction. The severity grading of AV patients was determined using the Global Acne Grading System (GAGS) [13], with a GAGS score of ≥ 19 classified as moderate to severe AV. Simultaneously, 20 healthy individuals, matched for gender and age with the patients, were recruited as the HC group. All participants signed an informed consent form agreeing to be included in the study and consented to the use of their demographic and clinical data in the scientific trial. This study has been approved by the Ethics Committee of the Affiliated Hospital of Southwest Medical University (No. KY2019139).

### 2.2 Experimental Process

Eighteen patients with moderate to severe AV were designated as the experimental group, referred to as the AV patients of MET Therapy (AVM) group. Additionally, 20 healthy individuals were classified as the control group, known as the HC group. The AVM group received MET hydrochloride sustained‐release tablets (500 mg, Qingdao Huanghai Pharmaceutical Co. Ltd., National Drug Standard H20040154), with one tablet administered twice daily for a duration of 12 weeks. During the treatment period, participants were prohibited from taking other lipid‐lowering medications, sex hormones, thyroxine, or any other drugs that could influence lipid metabolism. General demographic information, including age, gender, and BMI, of all enrolled participants was recorded prior to enrollment. Fasting venous blood samples were collected from all subjects at baseline to assess lipid levels, blood glucose (GLU), fasting insulin (INS), and other indicators, as well as to conduct serum lipidomics metabolism analysis. The GAGS scoring criteria were utilized to evaluate the facial lesions of patients with AV, while the VISIA‐CRTM imaging system (Canfield Scientific Inc., USA) was employed to determine the severity of skin lesions in these patients, including measurements for porphyrin and red areas (a higher measured value indicates less severe lesions) [14]. The AVM group underwent a follow‐up assessment after 12 weeks of treatment, during which the aforementioned procedures for sample detection and collection were repeated.

### 2.3 Collection and Detection of Serum Samples

A 2 mL fasting venous blood sample was collected upon entry using the Synchron LX‐20 automatic biochemical analyzer (Beckman Coulter, USA) to assess Glu, TG, TC, LDL, high‐density lipoprotein (HDL) and INS levels. INS levels were measured using an ELISA kit (Promega, USA). The Homeostasis Model Assessment of Insulin Resistance (HOMA‐IR) was calculated based on INS and GLU values to evaluate insulin resistance, using the formula HOMA‐IR = (fasting insulin × fasting blood glucose)/22.5. Changes in serum lipid metabolomics were analyzed using LC–MS.

### 2.4 Analysis of Serum Lipid Metabolomics

#### 2.4.1 Metabolomics Sample Preparation

The 200 μL serum samples were combined with 80 μL of methanol and 400 μL of methyl tert‐butyl ether (MTBE). The mixture was swirled and mixed for 30 s, followed by ultrasonic extraction for 30 min at 5°C and 40 kHz. The samples were then placed at −20°C for 30 min and subsequently centrifuged at high speed (18 900×*g*) for 15 min. Next, 350 μL of the supernatant was transferred to a vacuum concentrator to evaporate the solvent. After drying, 100 μL of the reconstitution solution (isopropanol: acetonitrile = 1:1) was added, and the mixture was vortexed for 30 s before being sonicated in an ice water bath for 5 min (40 kHz). Finally, the mixture was centrifuged at high speed (18 900×*g*) for 5 min, and 80 μL of the supernatant was transferred to a sample vial for analysis [15, 16].

#### 2.4.2 Chromatography‐Mass Spectrometry (LC–MS Analysis) Conditions

The instrument platform for this LC–MS analysis is the Thermo Scientific UPLC‐Q Exactive system, which features ultra‐high‐performance liquid chromatography coupled with tandem Fourier transform mass spectrometry. Chromatographic separation was performed using a BEH C18 column (100 mm × 2.1 mm i.d., 1.7 μm) at a column temperature of 40°C. The mobile phase consisted of a 10 mmol/L ammonium acetate solution in 50% acetonitrile (A, containing 0.1% formic acid) and a 2 mmol/L ammonium acetate solution in acetonitrile/isopropanol/water (10/88/2) (B, containing 0.02% formic acid), with a flow rate of 0.40 mL/min. The gradient elution was carried out as follows: from 0 to 4 min, the composition changed from 65% to 40% A and 35% to 60% B; from 4 to 12 min, it shifted from 40% to 15% A and 60% to 85% B; from 12 to 21 min, it transitioned from 15% to 0% A and 85% to 100% B; from 21 to 24 min, mobile phase A was maintained at 0% and mobile phase B at 100%; from 24.0 to 24.1 min, the composition changed from 0% to 65% A and from 100% to 35% B; from 24.1 to 28 min, mobile phase A was linearly maintained at 65% and mobile phase B at 35%. Mass spectrometry data were collected in both positive and negative ion spray voltage modes at 3000 V, with a mass detection range of 200–2000 m/z, an ion source heating temperature of 370°C, and a collision energy of 20–60 V.

### 2.5 Multivariate Statistical Analysis of Lipidomics

R software (Ver. R4.1.1), Partial Least Squares Discriminant Analysis (PLS‐DA), and Orthogonal Partial Least Squares Discriminant Analysis (OPLS‐DA) were utilized for multivariate statistical analysis to examine the overall differences in metabolic profiles between groups. Differential metabolites were identified based on the variable importance in projection (VIP) values derived from the OPLS‐DA model and the *p*‐values from Student's t‐test, with thresholds established at VIP ≥ 1 and *p* < 0.05. Pathway enrichment analysis was conducted using the Kyoto Encyclopedia of Genes and Genomes (KEGG) database (KEGG_V202.09.18).

### 2.6 Statistical Analysis

Statistical software SPSS 20.0 was utilized to analyze the research data. Measurement data were expressed as mean ± standard deviation (*x* ± *s*). The chi‐square test was employed to assess gender differences, while the Mann–Whitney *U* test was used to analyze differences between groups. Additionally, Spearman correlation analysis was conducted to examine the relationship between serum differential lipid metabolites and the severity index of acne. *p* < 0.05 was considered statistically significant.

## 3 Results

### 3.1 Comparison of General Data Between Groups

There were no statistically significant differences between the AV group and the HC group regarding gender (*p* = 0.825) and BMI (*p* = 0.65). Although the HC group was older than the AV group (*p* < 0.001), all participants met the age criteria specified in the inclusion guidelines. As indicated in Table 1, following treatment, the AV patients exhibited a significant reduction in GAGS scores after oral administration of MET (*p* < 0.001), with notable improvements in the red area (*p* < 0.001) and the Porphyrin index (*p* = 0.01). However, levels of TG, TC, HDL, LDL, GLU, INS and HOMA‐IR did not show significant differences (*p* > 0.05).

**TABLE 1:** Clinical data of HC and AV patients.

Factors	HC	AVM_0_	AVM_1_	*p*
Facial GAGS	—	28.78 ± 4.61	14.67 ± 3.51	< 0.001
Red area (%)	67.98 ± 17.30	33.11 ± 19.94	45.83 ± 21.87	< 0.001
Porphyrin (%)	75.27 ± 8.32	57.96 ± 22.18	76.17 ± 20.73	0.01
TC (mmol/L)	3.99 ± 0.46	3.83 ± 0.47	4 ± 0.64	0.496
TG (mmol/L)	0.95 ± 0.43	0.86 ± 0.24	1 ± 0.43	0.457
LDL (mmol/L)	2.30 ± 0.36	2.24 ± 0.57	2.44 ± 0.64	0.343
HDL (mmol/L)	1.43 ± 0.27	1.37 ± 0.31	1.3 ± 0.35	0.486
GLU (mmol/L)	4.59 ± 0.38	4.6 ± 0.22	4.66 ± 0.3	0.342
INS (pmol/L)	7.85 ± 2.98	7.12 ± 2.57	7.66 ± 2.75	0.681
HOMA‐IR	1.61 ± 0.67	1.50 ± 0.54	1.58 ± 0.55	0.752

Abbreviations: AVM_0_, before treatment with metformin in AV patients; AVM_1_, after treatment with metformin in AV patients; GAGS, Global Acne Grading System; GLU, blood glucose; HC, healthy controls; HDL, high density lipoprotein; HOMA‐IR, homeostasis model assessment for insulin resistance; INS, fasting insulin; LDL, low density lipoprotein; *p*, AVM_0_ vs. AVM_1_; TC, total cholesterol; TG, triglyceride.

### 3.2 Lipid Metabolomics Characterization

**FIGURE 1:**
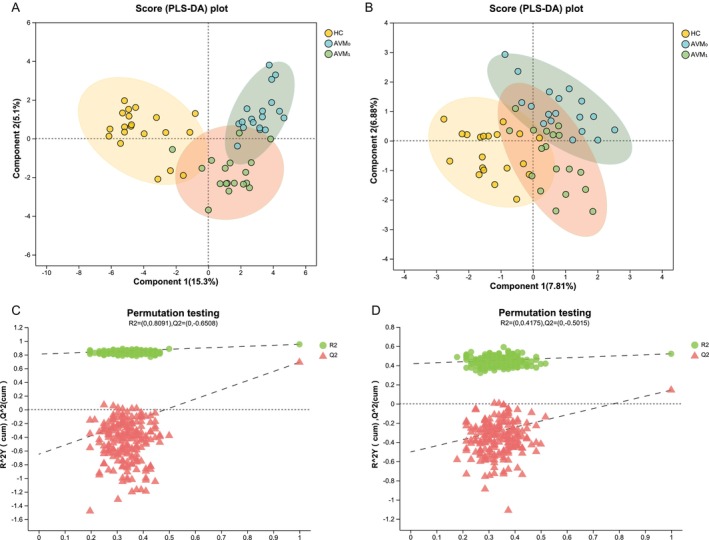
PLS‐DA analysis and permutation test of anionic and cationic models. (A) Cationic model score chart; (B) Anionic model score chart; (C) PLS‐DA cationic model permutation test; (D) Anionic model permutation test; R2Y (cum) and Q2 (cum) are model validation parameters, which represent model interpretability and model predictability, respectively. The cationic model coefficients of the sample in C were 0.404 (cum) for Q2 and 0.432 (cum) for R2Y. The anionic model coefficients of the sample in D were 0.145 (cum) for Q2 and 0.305 (cum) for R2Y, R2, and Q2 declined with the decrease of replacement retention, and the regression line showed an upward trend, indicating that there was no overfitting phenomenon in the model.

**FIGURE 2:**
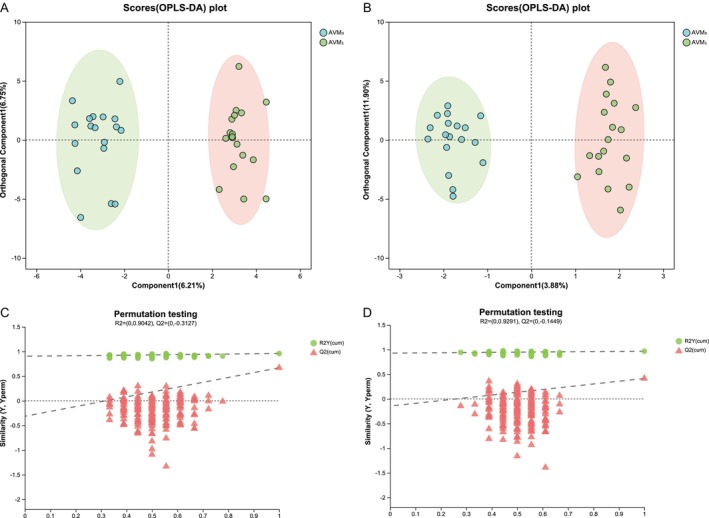
OPLS‐DA analysis and permutation test of anionic and cationic models. (A) Cationic model score chart; (B) Anionic model score chart; (C) OPLS‐DA cationic model replacement test; (D) OPLS‐DA anionic model replacement test. R2Y represents the explanation rate of the constructed model to the Y matrix, and R2Y (cum) represents the cumulative explanation rate. Q2 represents the prediction ability of the model, and the closer these two indicators are to 1, the model is more stable and reliable. The cationic model coefficients of the samples in C were 0.67 (cum) for Q2 and 0.96 for R2Y (cum). The coefficient of anionic model of the samples in D were 0.415 (cum) for Q2 and 0.968 (cum) for R2Y, indicating that the model has high prediction ability and good fitting degree.

We conducted LC–MS analysis to investigate the differences in lipid metabolism between AV patients and HC groups, as well as to evaluate the changes in lipid metabolism following oral administration of MET. In order to determine the lipid composition in serum more accurately, two detection modes of positive and negative ions were used in the analysis process, and the extraction method of samples and the detection conditions of chromatography and mass spectrometry were optimized [17, 18]. As the PLS‐DA analysis results showed, both cationic (Figure 1A) and anionic (Figure 1B) lipid metabolic profiles exhibited a clear separation trend between HC and AV patients. However, after MET treatment, the lipid metabolic profiles of AV patients gradually approached those of HC. To confirm the positive regulatory effect of MET on blood lipids, we established an OPLS‐DA model that filters out irrelevant noise and reduces result errors. Consistent with the PLS‐DA results, the AVM_0_ and AVM_1_ in the cationic (Figure 2A) and anionic (Figure 2B) models were distanced from each other, indicating significant differences in the serum lipid metabolic composition of AV patients before and after metformin treatment. Subsequently, we performed permutation tests on the cationic and anionic models in the PLS‐DA and OPLS‐DA analysis results to ensure the model's analytical capability. The regression lines and intercepts with the vertical axis in the results of Figures 1 and 2C,D were all < 0, indicating that the current model has good robustness, without overfitting issues, and possesses good fitting degree and predictive ability.

### 3.3 Differential Lipid Metabolites and Correlation Analysis

**FIGURE 3:**
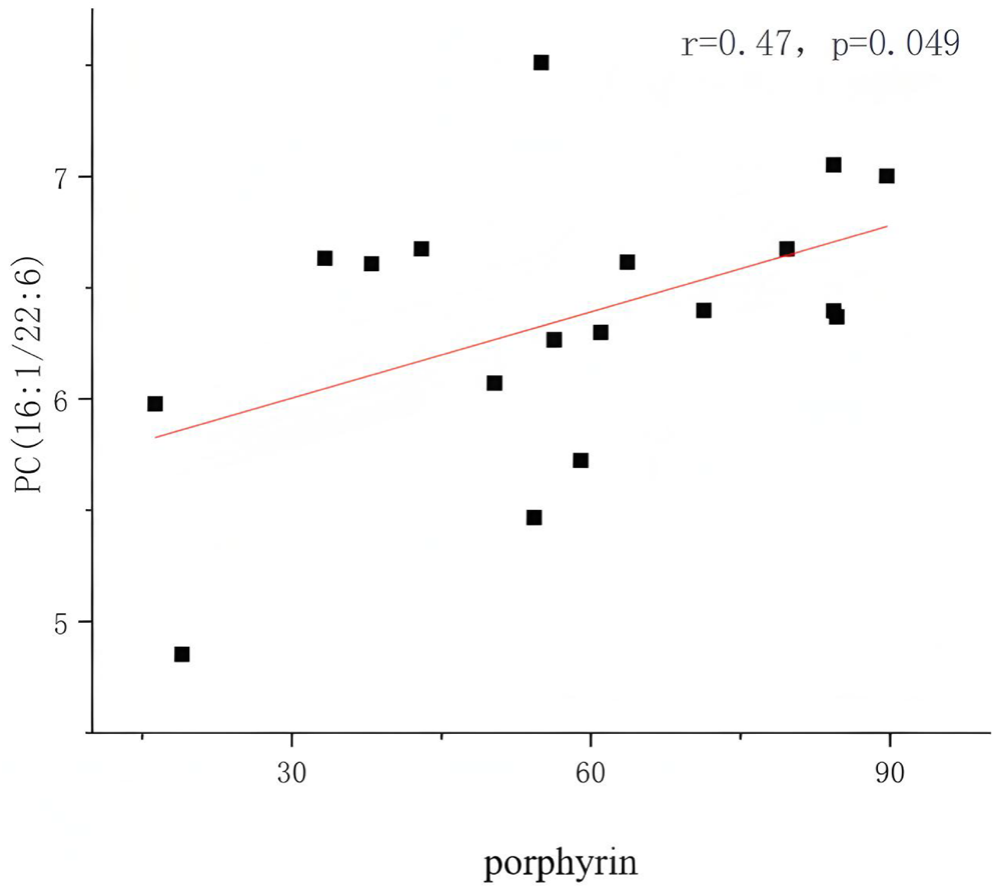
Scatter diagram of the relationship between lipid metabolite PC (16:1/22:6) and porphyrin. The horizontal coordinate is porphyrin, the vertical coordinate is PC (16:1/22:6).

A total of 258 lipid metabolites were detected in 38 serum samples by LC–MS analysis. According to the results of the OPLS‐DA model, 52 differential lipid metabolites that met the thresholds of VIP ≥ 1 and *p* < 0.05 were screened between HC and AV patients, as well as before and after MET treatment in AV patients. Table 2 presents the top 25 metabolites with the most significant differences along with their statistical values. Compared to HC, AV patients exhibited downregulation of 1 type of glycosylated ceramide (di‐hexosyl ceramide, Hex2Cer), 8 types of ceramides (Cer), 1 type of diacylglycerol (DG), 1 type of cephalin (LPE), 1 type of methylated phosphatidylcholine (MePC), 3 types of phosphatidylcholines (PC), 1 type of phosphatidylinositol (PI), and 1 type of phosphatidylethanolamine (PE) (*p* < 0.05). Additionally, there was an upregulation of 1 type of monogalactosyldiacylglycerol (MGDG), 2 types of lyso‐phosphatidylcholines (LPC), 1 type of methylated phosphatidylcholine (MePC), 2 types of phosphatidylcholine (PC), 1 type of sphingomyelin (SM), and 1 type of sphingosine (SPH) (*p* < 0.05). Notably, we observed that the aforementioned differential lipid metabolites in patients with AV returned to levels comparable to HC to varying degrees following oral administration of metformin (*p* < 0.05), which suggests that the lipid metabolism disorder in AV patients was significantly improved after the intervention. Subsequently, we conducted Spearman correlation analyses between the differential lipid metabolites listed in Table 2 and the severity indicators of acne, including the GAGS score, red area, and porphyrin area index, with the results presented in Figure 3. The serum concentration of PC (16:1/22:6) in AV patients prior to treatment was positively correlated with the porphyrin area index (*r* = 0.47, *p* = 0.049).

**TABLE 2:** Comparison of serum differential lipid metabolites between HC and AV patients before and after treatment.

Metabolites	HC	AVM_0_	AVM_1_	*p*_1_	*p*_2_
Hex2Cer (m17:0/22:6)	6.42 ± 0.31	6.17 ± 0.22	6.45 ± 0.26	0.005	< 0.001
MGDG (18:2/20:4)	7.83 ± 0.43	8.18 ± 0.28	7.63 ± 0.69	0.017	0.016
Cer (d16:0/12:0)	7.08 ± 0.12	6.72 ± 0.23	7.02 ± 0.08	< 0.001	< 0.001
Cer (d16:0/16:0)	9.3 ± 0.15	9 ± 0.2	9.32 ± 0.08	< 0.001	< 0.001
Cer (d16:0/18:0)	9.11 ± 0.16	8.8 ± 0.18	9.07 ± 0.08	< 0.001	< 0.001
Cer (d16:0/18:0 + O)	8.52 ± 0.16	8.19 ± 0.21	8.5 ± 0.08	< 0.001	< 0.001
Cer (t18:0/14:0)	6.87 ± 0.14	6.64 ± 0.15	6.97 ± 0.13	< 0.001	< 0.001
Cer (t18:0/16:0)	8.53 ± 0.16	8.2 ± 0.2	8.51 ± 0.08	< 0.001	< 0.001
Cer (t18:0/18:0)	8.19 ± 0.22	7.84 ± 0.32	8.24 ± 0.11	< 0.001	< 0.001
Cer (t18:0/18:0 + O)	7.44 ± 0.18	7.1 ± 0.23	7.45 ± 0.11	< 0.001	< 0.001
DG (20:5/18:2)	6.93 ± 0.46	6.38 ± 0.49	6.78 ± 0.28	0.001	0.014
LPC (18:1)	7.03 ± 0.17	7.19 ± 0.19	7 ± 0.19	0.019	0.009
LPC (20:2)	6.94 ± 0.37	7.38 ± 0.19	7.11 ± 0.19	< 0.001	< 0.001
LPE (16:0)	7.07 ± 0.14	6.85 ± 0.09	6.97 ± 0.15	< 0.001	0.008
MePC (16:0/18:1)	6.77 ± 0.57	7.27 ± 0.22	6.99 ± 0.34	0.002	0.01
MePC (18:3e/22:6)	8.07 ± 0.18	7.81 ± 0.18	8.05 ± 0.12	0.001	< 0.001
PC (8:0e/11:3)	6.72 ± 0.25	6.35 ± 0.4	6.71 ± 0.25	< 0.001	0.002
PC (6:0/22:6)	6.22 ± 0.18	7.05 ± 0.27	6.66 ± 0.16	< 0.001	< 0.001
PC (18:2e/20:2)	7.33 ± 0.55	8.07 ± 0.32	7.75 ± 0.2	< 0.001	0.001
PC (16:1/22:6)	6.91 ± 0.41	6.37 ± 0.61	6.89 ± 0.4	0.002	0.004
PC (20:4/22:6)	8.19 ± 0.26	7.7 ± 0.22	8.14 ± 0.16	< 0.001	< 0.001
PE (18:0p/22:6)	8.53 ± 0.18	8.2 ± 0.19	8.39 ± 0.1	< 0.001	0.002
PI (18:0/20:4)	8.7 ± 0.09	8.5 ± 0.07	8.59 ± 0.09	< 0.001	0.002
SM (d18:2/20:3)	6.68 ± 0.45	7.36 ± 0.2	6.95 ± 0.44	< 0.001	0.002
SPH (t16:0)	7.89 ± 0.12	8.16 ± 0.27	7.78 ± 0.11	< 0.001	< 0.001

Abbreviations: Cer, ceramide; DG, diacylglycerol; Hex2Cer, dihexosyl ceramide; LPC, lysophosphatidylcholine; LPE, cephali; MePC, methylated phosphatidylcholine; MGDG, monogalactosyldiacylglycerol; *p*_1_, HC vs. AVM_0_; *p*_2_, AVM_0_ vs. AVM_1_; PC, phosphatidylcholine; PE, phosphatidylethanolamine; PI, phosphatidylinositol; SM, sphingomyelin; SPH, sphingosine.

### 3.4 KEGG Enrichment Analysis of Differential Metabolites Before and After MET Treatment

**FIGURE 4:**
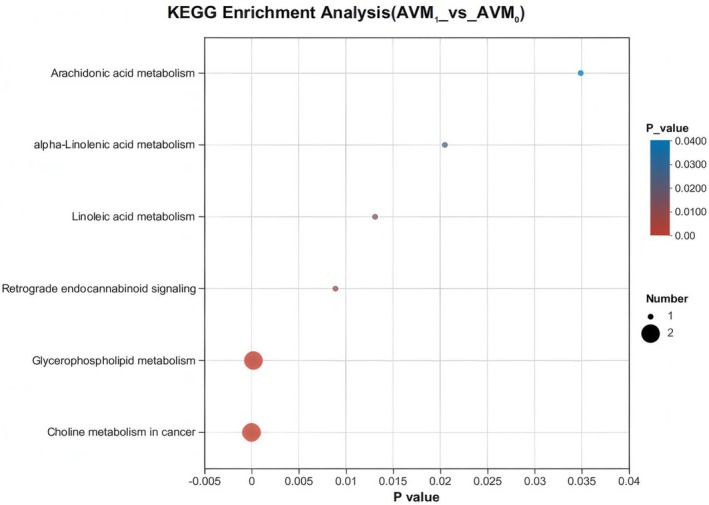
KEGG enrichment analysis bubble map in AV and HC. The abscissa is the enrichment significance *p* value, and the *p* value < 0.05 is considered as a significant enrichment term. Ordinate is KEGG pathway; the color of bubbles from red to purple indicates that the *p* value decreases in turn. The larger the bubble, the more metabolites are enriched in the pathway.

We imported the various lipid metabolites from the AVM_0_ and AVM_1_ groups, as shown in Table 2, into the KEGG database for metabolic pathway enrichment analysis. Ultimately, we identified six metabolic pathways with significant differences: arachidonic acid metabolism, alpha‐linolenic acid metabolism, linoleic acid metabolism, glycerophospholipid metabolism, retrograde endocannabinoid signaling, and choline metabolism in cancer. All pathways were downregulated following MET treatment, with the most pronounced enrichment observed in the choline metabolism pathway in cancer (Figure 4).

## 4 Discussion

Based on the current understanding of the pathogenesis of AV, the medications used to treat moderate to severe AV primarily target the infection caused by *Cutibacterium acne*, the regulation of keratinization in the sebaceous gland duct epithelium, and the reduction of sebum secretion [19]. However, isotretinoin, which is the first‐line treatment for moderate to severe AV, has been limited in clinical application to some extent due to its side effects, including dryness of the skin and mucous membranes, elevated blood lipids, reduced insulin sensitivity, and teratogenicity [20, 21]. Previous studies, along with our preliminary experiments, have indicated that disorders of serum lipid metabolism and insulin resistance in patients with AV may also be significant preconditions contributing to the onset and progression of the disease [3, 22]. In recent years, MET has been shown to enhance insulin sensitivity and positively regulate lipid metabolism. Using a mouse model that mimics human‐like lipoprotein metabolism (APOE*3‐Leiden.CEPT), led to the discovery that MET could reduce the plama level of TG and very‐low‐density lipoprotein (VLDL) without affecting the production of VLDL‐TG in the liver [7]. Similar findings have emerged from related clinical trials, indicating that elderly patients undergoing metformin treatment exhibited increased HDL levels in serum, while TC and LDL levels were downregulated [23]. However, it remains unclear whether MET exerts its therapeutic effects in AV patients by regulating lipid metabolism. The results of this study indicated that AV patients did not exhibit insulin resistance before or after MET treatment [11, 12]. Furthermore, the intervention with MET significantly improved the severity of AV in patients, but there were no significant changes in lipid levels. This lack of change may be attributed to the small sample size of the study and the limited sensitivity of the clinical testing methods. To address this, we employed LC–MS, a more robust method for metabolomic analysis, to further investigate the effects of MET on lipid metabolism in AV patients.

In the subsequent analysis, we found that MET had a significant positive regulatory effect on serum lipid metabolomics in patients with AV. Notably, PC, identified among the differential lipid metabolites, is a key intermediate in the glycerophospholipid metabolic pathway [24]. This metabolite plays a crucial role in maintaining the homeostasis of this pathway. Additionally, its unique solubility, in the presence of bile salts, breaks down the almost insoluble cholesterol into mixed micro‐granules, thereby reducing cholesterol absorption [25, 26]. Secondly, studies have reported that in mice, dietary supplementation with PC in vivo could inhibit cholesterol absorption, increase the excretion of cholesterol in feces, and thereby lower the cholesterol level in plasma [27]. Furthermore, PC (16:1/22:6) can inhibit the release of inflammatory factors by enhancing PPAR‐γ activity, which downregulates the TLR‐4/NF‐κB and c‐Jun N‐terminal kinase (JNK) pathways [28, 29]. This anti‐inflammatory property has been demonstrated in patients with non‐alcoholic fatty liver disease, where serum levels of PC (16:1/22:6) positively correlate with changes in HDL‐C concentrations and negatively correlate with changes in the inflammatory mediators IL‐1β and TNF‐α [30]. In addition, during the serum lipid metabolism process, PC can be hydrolyzed by cytosolic phospholipase A2 (cPLA2) to generate LPC in a recycling pathway known as the “Lands cycle” [31], which is an inflammatory lipid found to be upregulated in the expression of various inflammation‐related diseases [32]. Research has demonstrated that pro‐inflammatory processes, such as the release of chemokines and the production of reactive oxygen species, are associated with elevated serum levels of saturated and monounsaturated LPCs, including LPC (18:1) [33]. In our study, we observed that MET intervention led to a gradual recovery of serum lipid metabolites LPC (18:1) and PC (16:1/22:6) to levels comparable to those of HC in patients with AV. Furthermore, we found a positive correlation between the latter and the AV porphyrin index. Therefore, we propose that MET may exert anti‐inflammatory effects by positively regulating lipid metabolism.

In KEGG analysis, we discovered that the choline metabolic pathway in cancer is significantly downregulated following MET treatment. Acetylcholine (ACh), a well‐known molecule in the neurotransmitter family, can be expressed in basal supra‐sebaceous sebocytes and non‐neuronal tissue cells, such as those in the skin, via muscarinic and nicotinic receptors [34], respectively [35, 36]. Research has demonstrated that the topical application of anticholinergic drugs can inhibit lipid synthesis in sebaceous gland cells [37], reduce sebum secretion, and subsequently alleviate the issue of enlarged pores in patients [36, 38]. These clinical findings have prompted researchers to pay closer attention to the relationship between AV and the cholinergic system. Cartlidge et al. found that patients with mild acne who received a continuous 4‐week treatment of topical anticholinergic drugs experienced a significant reduction in sebum production in their forehead skin [39]. Our results indicated that after MET intervention in patients with moderate to severe AV, not only was the severity alleviated, but there was also a downward trend in choline metabolism. This may be related to the positive regulation of lipid metabolism in patients receiving MET. The active intervention of the drug appears to enhance the cholinergic system, helping to prevent excessive sebum secretion. However, the specific metabolites and molecular mechanisms underlying these changes require further investigation. Additionally, the downregulated metabolic pathways of arachidonic acid and alpha‐linolenic acid following MET intervention are significant factors in the occurrence and progression of AV. Notably, alpha‐linolenic acid is considered a possible participant in follicular hyperkeratosis [40, 41], which is crucial in the formation of papules in patients with AV [41]. Furthermore, Alestas et al. [42] proposed that the oxygenase activity of 5‐lipoxygenase (LOX) converts arachidonic acid into a substrate for leukotriene synthesis, which is a potent chemotactic mediator that induces the production of inflammatory mediators by neutrophils, thereby triggering an inflammatory response.

## 5 Conclusions

In conclusion, we believe that the therapeutic effect of MET on patients with AV may be achieved by regulating lipid metabolism and subsequently downregulating the levels of inflammatory mediators. Additionally, there is no clear correlation between insulin resistance and AV patients; the specific molecular mechanisms require further experimental verification. Moreover, this study has potential limitations, including a relatively small sample size and a lack of comprehensive, in‐depth analysis of lipidomics. Future research should further explore the roles of metabolic pathways and other molecular mechanisms to provide new insights into the occurrence, development, and treatment of AV.

## Author Contributions

Jie Hu was involved in writing the original draft, analysis, and interpretation of data. Yuxin He revised it critically for important intellectual content. Peiyang Du was involved in collecting resources. Yongqiong Deng was responsible for supervision, project administration. Xia Xiong was involved in supervision and funding acquisition. Yaxin Huang gave final approval of the version to be published.

## Ethics Statement

The studies involving human participants were reviewed and approved by the Ethics Committees of the hospital affiliated with Southwest Medical University (no: KY2019139).

## Conflicts of Interest

The authors declare no conflicts of interest.

## Data Availability

The data that support the findings of this study are available on request from the corresponding author.
